# PERCEIVED JOB DISCRIMINATION AND SLEEP HEALTH AMONG WORKING WOMEN: FINDINGS FROM THE SISTER STUDY

**DOI:** 10.1093/geroni/igz038.2863

**Published:** 2019-11-08

**Authors:** Soomi Lee, Anne-Marie Chang, Dale P Sandler, Orfeu M Buxton, Chandra L Jackson

**Affiliations:** 1 University of South Florida, Tampa, Florida, United States; 2 Pennsylvania State University, State College, Pennsylvania, United States; 3 NIEHS, Durham, North Carolina, United States; 4 National Institutes of Health, Durham, North Carolina, United States

## Abstract

Job discrimination is a social stressor that may lead to sleep health disparities in workers; however, limited research has examined the relationship, especially with specified sources of job discrimination. Using longitudinal data from the Sister Study, we tested the associations of perceived job discrimination (due to race, sex, age, and health conditions) with sleep health among working women (n=26,085). Among those without sleep difficulty at Time 1, race- and age-specific job discrimination was associated with increased odds of new onset sleep difficulty at Time 2. Moreover, among those without excessive sleepiness at Time 1, sex-, age-, and health-specific job discrimination predicted new onset of excessive sleepiness at Time 2. There was no association with sleep duration. We also found a dose-response relationship such that those who experienced job discrimination due to ≥3 reasons had greater odds of developing a sleep problem. Results suggest sleep health disparities emanating from the workplace.

